# Parental control and support for physical activity predict adolescents’ moderate to vigorous physical activity over five years

**DOI:** 10.1186/s12966-021-01107-w

**Published:** 2021-03-22

**Authors:** Radhouene Doggui, François Gallant, Mathieu Bélanger

**Affiliations:** 1 Department of family medicine, Université de Sherbrooke, Sherbrooke, Canada; 2 Centre de formation médicale du Nouveau-Brunswick, Université de Sherbrooke, Moncton, NB Canada; 3 Vitalité Health Network, Moncton, Canada

**Keywords:** Social support, Social influence, Adolescence, Youth, Physical activity

## Abstract

**Background:**

Social factors are important determinants of youth physical activity (PA), but the longitudinal association between parental behaviours and adolescent PA has not been clearly assessed. This prospective study examined average and lagged associations between perceived parental support and control with adolescents’ moderate to vigorous PA (MVPA); and assessed the independent associations between specific parental support and control behaviours and adolescents’ MVPA.

**Methods:**

Data from three cycles of the MATCH study, when 374 participants were 12, 16 and 17 years old, were included in this analysis. At each cycle, participants self-reported questionnaires on perceived tangible parental support, intangible support, and control behaviours as well as number of days per week attaining at least 60 min of MVPA. Mixed effect models were used to assess the longitudinal relationship between parental behaviours and MVPA. Cross-lagged panel design was used to assess the association of parental behaviours during early adolescence with MVPA during late adolescence.

**Results:**

Overall parental support (coef. = 0.46, *P* < 0.0001), tangible support (coef. = 0.37, *P* < 0.0001), encouragement (coef. = 0.12, *P* = 0.025) and transportation (coef. = 0.25, *P* < 0.0001) were positively associated with MVPA, whereas parental control was a negative predictor of MVPA (coef. = − 0.18, *P* = 0.003). Perceived parental behaviours appeared to have long term associations (5 y.) with MVPA as parent support (coef. = 0.40, *P* = 0.006) and co-participation (coef. = 0.33, *P* = 0.017) reported around age 12 were positively associated with MVPA measured 5 years later.

**Conclusions:**

Parental support for PA, particularly in the form of tangible support, may be a key factor to include in interventions aiming to promote PA during adolescence. In contrast, parents should be encouraged to avoid control behaviours as these appear to lead to lower MVPA among adolescents.

## Introduction

Physical activity (PA) plays an important role in normal growth and development of youth and is a predictor of their health [1]. Only about 1 in 3 young Canadians attain the recommended weekly average of at least 60 min of daily moderate-to-vigorous PA (MVPA) [2]. The childhood to adolescence transition represents a period of individual development often marked by a decline in MVPA levels [3]. Throughout this period, the influence of various behavioural determinants may change as individuals gain control and autonomy [4]. Since adolescence is associated with the development of stronger social affiliations outside of the family network, it is possible that the effect of the family environment changes during this period. Still, parental support for PA can generally be perceived as social support, which is thought to promote PA through better self-efficacy [5]. The social cognitive theory suggests that participation in PA is promoted through self-efficacy because of an enhanced capacity to make abstraction of potential barriers to PA participation [6]. Social support could also have a positive influence on individuals’ motivation for PA, which in turn would influence behaviour [7].The influence of parents on adolescents’ PA levels nevertheless remains ambiguous as most studies to date concentrated on aggregated parental behaviours without accounting for possible overlapping between them [8, 9].

Notwithstanding limitations of previous studies, results to date suggest that parents can regulate offspring’s participation in PA through verbal support, co-participation, provision of resources, encouragement, guided choices, involvement and offering of rewards [8, 10–12]. Studies document positive associations between parental support and PA among adolescents [13, 14]. Parental support behaviours relating to services facilitating PA (e.g. transportation) can be categorized as tangible whereas providing verbal encouragements and praises in relation to PA can be categorized as intangible support [12, 15]. Although tangible and intangible parental support may have different associations with MVPA, most studies present parental support as a cumulative score combining all parental support behaviours [8]. Further, studies to date do not fully assess whether associations between parental support behaviours and MVPA are sustained throughout adolescence. As adolescents seek to gain autonomy, it is possible that the influence of intangible parental support declines faster with age than the influence of tangible support, which adolescents continue to depend on to address external barriers to physical activity participation, including provision of transportation, equipment and registration costs [16]. Tangible parental support may also continue to have a positive influence on adolescents’ physical activity if they depend on the participation of parents to pursue participation in the activity themselves.

Beyond parental support, parenting styles may also influence youth’s participation in PA. Parenting style refers to the emotional and relational climate created by parents and is a combination of emotional involvement (warmth) and demandingness (control) [17]. Ambiguity exists in relation to the direction of an association between parenting style and PA [17, 18]. Whereas a cross-sectional study among children 10 to 11 years showed that low parental control is positively associated with children’s PA [19], a longitudinal-study showed that parental control was positively associated with PA 3 years later among elementary school students [20]. It is also unclear if parental control is linked to adolescents’ MVPA.

Because social determinants represent a large proportion of PA socio-ecological frameworks and adolescence is a period when parents may still exert influence on their offspring’s MVPA, it is essential to gain a better understanding of the influence specific parental behaviours and parenting styles may have on MVPA throughout adolescence. Such knowledge could help inform interventions among parents to promote physically active lifestyles among adolescents. Therefore, this study aimed to 1) examine longitudinal and lagged associations between total, tangible and intangible parental support and control behaviours for PA on the MVPA of adolescents; and 2) assess the independent associations between various parental support practices (i.e. co-participation, transportation, motivational, encouragement, informational and modeling) and control (i.e. nagging and ordering) with adolescents’ MVPA.

### Methodology

#### Participants

The Monitoring Activities of Teenagers to Comprehend their Habits (MATCH) study [21], is an ongoing prospective study designed to describe the natural development of PA patterns of youth and identify their determinants. The MATCH study was designed to include a mix of students from schools from low, middle, and higher socioeconomic status in a variety of urban, suburban, and rural settings in French and English regions of New Brunswick. Briefly, in 2011, 806 participants were recruited from 17 schools across the province of New Brunswick, Canada when they were in Grade 5 or 6 (age 10–12 years). Participants were invited to fill three self-report questionnaires per year (every 4-months) until they completed Grade 12 (up to 24 survey cycles). The sample increased to 937 as other students from participating schools were allowed to enter the study in follow-up survey cycles. With a loss-to-follow-up proportion of less than 9% per year, 497 participants were still actively involved in the study in their last year of high school (Grade 12). On average, participants were followed up for 2.6 cycles and the most common reasons for losses to follow-up were having moved (*n* = 174), the school interrupting its participation (*n* = 191) and choosing to leave the study (*n* = 76). For the current analysis, we used data from cycles 9 (*n* = 617), 19 (*n* = 367) and 22 (*n* = 208), (when participants were approximately 12, 16 and 17 years, respectively) since information on perceived parental support and control behaviours was captured only in these cycles. Participants who participated in at least two of the three cycles which included measures of parental behaviours were included in this analysis.

### Measures

#### Moderate to vigorous PA

Participants self-reported their involvement in MVPA at each cycle using a two-item questionnaire [22]. Specifically, participants were provided a definition and examples of MVPA and then asked to indicate the number of days they engaged in at least 60 min of MVPA in 1) the past week and 2) the typical week. Response options ranged from 0 to 7 and the average of the two items showed good test-retest reliability with an intraclass correlation (ICC) of 0.77 when tested among 138 adolescents aged 12.1 (standard deviation = 0.9) years [22]. The MVPA score obtained by averaging the two items also correlated with measures of accelerometers accumulated over 5 to 7 days (*r* = 0.40, *p* < 0.001) [22]. To account for seasonal variation in MVPA, the current analysis uses the average of participants’ MVPA scores in all three survey cycles of a given school year.

#### Perceived parental support

Participants reported perceived parental support at cycles 9 (age 12), 19 (age 16) and 22 (age 17) through 5 components of support for each of their parents using the Parental Support Scale [23]. The 5 components represent parental *co-participation* (Did your father (mother) participate in PA or play sports with you?), *transportation* (Did your father (mother) bring you to a place where you can do PA or play sports?), *watching* (Did your father (mother) watch you do PA or play sports?), *encouragement* (Did you father (mother) encourage to practice PA?) and *informational* (Did your father (mother) tell you that PA is good for your health?). An additional item was added to capture modeling (Did your father (mother) do any PA or participate in sports?). For each item, scoring ranged from 1 (never/none) to 5 (nearly everyday). A tangible support score was computed by averaging items representing co-participation, transportation, and watching. Similarly, items representing encouragement, informational (advising) and modeling were averaged to represent intangible support. Both scores could range from 1 to 5, where higher scores reflect supportive parental practices. Average scores obtained for both parents was used for analyses. Two-week test-retest reliability of the scale had an ICC of 0.88 and a previous study demonstrated that youth’s responses on this scale are correlated (*r* = 0.6, *p* < 0.001) with responses from parents [23]. Scale reliability in the current study was good (Cronbach’s alpha = 0.84).

#### Perceived parental control

Participants reported their perception of parental control at cycle 9 (age 12), 19 (age 16) and 22 (age 17) through one item representing ordering and one item representing nagging [24] for each of their parents. Specifically, participants responded to: “*Did your father (mother) order you to do sports or PA?*”, “*Did your father (mother) annoy you to do sports or PA?*”. For each item, scores ranged from 1 (never) to 5 (very often). Higher average scores capture perceptions of higher parental control. Internal consistency of scores in the current study was good (Cronbach’s alpha = 0.86) and a previous study supported the construct validity of this scale with reports of it being negatively correlated with measures of self-efficacy and enjoyment of physical activity [15].

#### Covariates

Participants reported their gender and postal code, which was used to obtain their neighborhood average income, drawn from the 2011 National Household Survey census data expressed as tertiles herein. Schools were selected to represent living area (i.e. rural or urban) and cultural backgrounds (i.e. French or English language) [21].

### Statistical analysis

Since missing data for MVPA and parental parameters affected less than 10% of the sample and Little’s test suggested that missing data were missing completely at random (*P* = 0.64), we considered missing data as inconsequential and did not pursue imputation. Complete cases for MVPA and perceived parental parameters were used in the analyses. To examine associations between parental support and MVPA over time, two-level linear mixed models accounting for clustering due to repeated measures were used. We also examined whether within-person variation in the outcome followed a linear or quadratic time trend. The quadratic term was not significant, so we did not retain it for subsequent models. In initial crude analyses, we modeled MVPA as a function of total support, tangible and intangible support, and parental control separately. Then, in partially adjusted models, MVPA was modeled as a function of each parental parameter with adjustments for time and covariates. A fully adjusted model was then computed by including all parental support and control variables and potentially confounding variables described above into one model. To test if the estimated relationship of parental support and control behaviours on MVPA changed as participants aged, we included interaction terms (e.g. parental support x age and parental control x age).

Similarly, a series of partially adjusted models and one fully adjusted model were computed to assess the independent association of each component of parental support behaviours (co-participation, transportation, watching, encouragement, informational and modeling) with MVPA. For each model above, we used an unstructured within-individual correlation structure as no prior assumption about the correlation was presumed and time points were unequally spaced for the measures of parenting. Because of previously documented gender differences in MVPA and perceived parental support [9], we tested interaction terms for moderation effects of parental support and parent control on MVPA by gender. These were not significant, so analyses were not stratified by gender. We used the Wald test to compare slopes associated with each individual form of parental support [25]. Orthogonal polynomial contrasts were used to test MVPA across different covariates for linear trends [26]. All models were carried out using *mixed* Stata command and were fitted using the REML method (significance level of α < 0.05).

To examine lagged associations of parental support and control on adolescents’ future MVPA, we used a cross-lagged panel design (*sem* Stata command) by following the crude, partially adjusted and fully adjusted sequence described above. In these models, we also adjust the estimate of the lagged variable effect for the dependent variable at the previous time point [27]. Because the lagged-response model is recommended for use with approximately equally spaced time intervals between measurements, we built two separate sets of models (set 1: 12y vs. 16y and 12y vs. 17y; set 2: 16y vs. 17y). We used the full information maximum likelihood method and goodness of fit was assessed based on the Chi-square test (*P* > 0.05), root mean square error of approximation (RMSEA< 0.06), comparative fit index (CFI > 0.95), and Tucker-Lewis index (TLI > 0.95) [28].

## Results

A total of 436 youth participated in at least two of the three survey cycles of interest. Of these, 374 reported their level of MVPA and perception of parental behaviours and were retained for the analyses. Participants retained and participants excluded from the analyses were not different from each other with regards to gender distribution (*P* = 0.19), age (*P* = 0.32), or physical activity level at study inception (*P* = 0.94). Mean (standard deviation) age of participants at cycle 9 (first survey cycle for the current analyses) was 12.5 (0.6) years (Table 1), which coincides with the first stage of adolescence life in accordance with the World Health Organization [29]. Both parental support (mean values: 3.4, 2.7, and 2.8 at age 12, 16 and 17, respectively; *P* = 0.007) and control (mean values: 1.9, 1.5, and 1.4 at age 12, 16 and 17, respectively; *P* < 0.0001) showed a linear decline over time. The number of days per week participants reported taking part in at least 60 min of MVPA decreased from 5.8(1.5) when they were 12 years to 4.9 (2.0) when they were 17 years old.

**Table 1:** Sociodemographic characteristics of participants retained for the analyses (*n* = 374)

	n	% or mean (sd)
**Gender**		
Girls	220	58.8
Boys	154	41.2
**Age**		
Age at the first cycle of analysis	374	12.5 ± 0.6
**Population density**		
Rural	191	51.1
Urban	183	48.9
**Neighborhood income (CAD $)**		
Low (15487–27,698)	131	35.0
Moderate (27777–34,796)	129	34.5
High (35284–57,098)	114	30.5
**Language**		
French	280	74.9
English	94	25.1

In crude analyses, higher parental support was associated with higher levels of MVPA, whereas higher parental control was associated with lower MVPA (Table 2). These trends were sustained in partially and fully adjusted analyses. In fully adjusted models, a one-unit increase in parental support was associated with 0.46 more active days per week. In contrast, a one-unit increase in parental control was associated with 0.18 fewer active days per week. In a head-to-head comparison of parental behaviours and styles, parental support emerged as a more important determinant of change in MVPA than the coefficient representing parental control (*P* < 0.0001).

**Table 2:** Associations among parental support, parental control and number of days reporting at least 60 minutes of moderate to vigorous physical activity

	Number of days reporting at least 60 minutes of moderate to vigorous physical activity									
	Crude analysis^**a**^		Partially adjusted analysis^**b**^				Fully adjusted analysis^**c**^		Fully adjusted analysis^**c**^ with interactions	
	Coef.^**d**^	95% C.I.^**e**^	Coef.^**d**^	95% C.I.^**e**^	Coef.^**d**^	95% C.I.^**e**^	Coef.^**d**^	95% C.I.^**e**^	Coef.^**d**^	95% C.I.^**e**^
	**Total parental scores**									
Parental control	0.09	-0.03 – 0.21			-0.05	-0.16 – 0.06	-0.18	-0.30 – -0.06	0.14	-0.07 – 0.02
Parental control x age	–	–	–	–	–	–	–	–	-0.02	-0.07 – 0.02
Parental support	0.41	0.29 – 0.52	0.42	0.31 – 0.53			0.46	0.35 – 0.58	-0.15	-0.80 – 0.48
Parental support x age	–	–	–	–	–	–	–	–	0.04	-0.001 – 0.08
	**Tangible vs intangible support**									
Tangible support score	0.39	0.29 – 0.49	0.40	0.29 – 0.50	–	–	0.37	0.22 – 0.51	-0.05	-0.66 – 0.56
Tangible support score x age	–	–	–	–	–	–	–	–	0.03	-0.01 – 0.07
Intangible support score	0.30	0.19 – 0.40	0.30	0.20 – 0.41	–	–	0.10	-0.40 – 0.13	-0.60	-1.22 – 0.02
Intangible support score x age	–	–	–	–	–	–	–	–	0.05	0.01 – 0.09
	**Individual behaviours of support**									
Encouragement	0.23	0.14 – 0.30	0.23	0.15 – 0.31	–	–	0.12	0.02 – 0.23	-0.25	-0.77 – 0.26
Encouragement x age	–	–	–	–	–	–	–	–	0.03	-0.01 – 0.19
Informational	0.19	0.11 – 0.27	0.17	0.10 – 0.26	–	–	-0.03	-0.13 – 0.08	-0.33	-0.84 – 0.17
Informational x age	–	–	–	–	–	–	–	–	0.02	-0.01 – 0.05
Modeling	0.13	0.05 – 0.21	0.12	0.03 – 0.21	–	–	0.03	-0.07 – 0.13	-0.43	-0.93 – 0.06
Modeling x age	–	–	–	–	–	–	–	–	0.03	-0.002 – 0.06
Co-participation	0.14	0.05 – 0.23	0.13	0.04 – 0.23	–	–	-0.03	-0.14 – 0.08	-0.30	-0.85 – 0.25
Co-participation x age	–	–	–	–	–	–	–	–	0.02	-0.02 – 0.06
Transportation	0.32	0.24 – 0.40	0.31	0.23 – 0.39	–	–	0.25	0.15 – 0.36	-0.52	-0.55 – 0.44
Transportation x age	–	–	–	–	–	–	–	–	0.02	-0.01 – 0.05
Watching	0.27	0.18 – 0.35	0.25	0.17 – 0.34	–	–	0.08	-0.03 – 0.20	0.14	-0.35 – 0.63
Watching x age	–	–	–	–	–	–	–	–	0.00	-0.03 – 0.03
	**Individual behaviours of control**									
Nagging	-0.05	-0.15 – 0.04	–	–	-0.06	-0.16 – 0.04	-0.13	-0.26 – -0.01	0.06	-0.51 – 0.64
Nagging x age	–	–	–	–	–	–	–	–	-0.01	-0.05 – 0.03
Ordering	-0.02	-0.13 – 0.09	–	–	-0.04	-0.15 – 0.07	-0.05	-0.18 – 0.09	0.34	-0.29 – 0.97
Ordering x age	–	–	–	–	–	–	–	–	-0.03	-0.18 – 0.31

^a^Univariate analysis

^b^Adjustment for time, gender, age, urban/rural, neighborhood income and language

^c^Adjustment for time, gender, age, urban/rural, neighborhood income, language, parental support and control variables

^d^Crude or adjusted slope coefficient

^e^95% CI confidence interval for crude or adjusted coefficient

In crude and partially adjusted models, both tangible and intangible forms of parental support were positively associated with MVPA, and both forms of parental support also appeared to have similar level of association with MVPA of adolescents (Table 3). In the fully adjusted model however, only tangible support emerged as positively associated with MVPA.

**Table 3:** Head-to-head comparison of the different forms of control and support predictability for the difference in number of days reporting at least 60 min of moderate to vigorous physical activity

Parental support						
	Tangible vs. intangible support (*P*-values)					
	*Crude analysis*					
Tangible support	–					
Intangible support	*P*^a^ = 0.68					
	*Partially adjusted analysis*^b^					
Tangible support	–					
Intangible support	*P*^a^ = 0.66					
	*Full adjusted analysis (with interaction term)* ^c^					
Tangible support	–					
Intangible support	*P*^a^ = 0.0040					
	Individual behaviours comparison (*P*-values)					
	Informational	Modeling	Co-participation	Transportation	Watching	
	*Crude analysis*					
Encouragement	*P*^a^ = 0.97	*P*^a^ = 0.33	*P*^a^ = 0.34	*P*^a^ = 0.35	*P*^a^ = 0.75	
Informational	–	*P*^a^ = 0.30	*P*^a^ = 0.29	*P*^a^ = 0.35	*P*^a^ = 0.77	
Modeling	–	–	*P*^a^ = 0.93	*P*^a^ = 0.051	*P*^a^ = 0.18	
Co-participation	–	–	–	*P*^a^ = 0.067	*P*^a^ = 0.22	
Transportation	–	–	–	–	*P*^a^ = 0.55	
	*Partially adjusted analysis*^b^					
Encouragement	*P*^a^ = 0.96	*P*^a^ = 0.33	*P*^a^ = 0.36	*P*^a^ = 0.36	*P*^a^ = 0.75	
Informational	–	*P*^a^ = 0.29	*P*^a^ = 0.31	*P*^a^ = 0.36	*P*^a^ = 0.77	
Modeling	–	–	*P*^a^ = 0.96	*P*^a^ = 0.050	*P*^a^ = 0.18	
Co-participation	–	–	–	*P*^a^ = 0.072	*P*^a^ = 0.23	
Transportation	–	–	–	–	*P*^a^ = 0.55	
	*Full adjusted analysis*^c^					
Encouragement	*P*^a^ = 0.13	*P*^a^ = 0.24	*P*^a^ = 0.069	*P*^a^ = 0.062	*P*^a^ = 0.76	
Informational	–	*P*^a^ = 0.57	*P*^a^ = 0.77	*P*^a^ = 0.0007	*P*^1^ = 0.23	
Modeling	–	–	*P*^a^ = 0.46	*P*^a^ = 0.0019	*P*^a^ = 0.41	
Co-participation	–	–	–	*P*^a^ = 0.0003	*P*^a^ = 0.13	
Transportation	–	–	–	–	*P*^a^ = 0.071	
**Parental control behaviours (***P*-values)						
	*Crude analysis*					
Nagging	*P*^a^ = 0.78					
Ordering						
	*Partially adjusted analysis*^b^					
Nagging	*P*^a^ = 0.89					
Ordering						
	*Full adjusted analysis*^c^					
Nagging	*P*^a^ = 0.46					
Ordering						

^a−^ Crude or adjusted P-value for slope coefficients comparison by test of Wald

^b^ Adjustment for time, gender, age, urban/rural, neighborhood income and language

^c−^ Adjustment for time, gender, age, urban/rural, neighborhood income, language, parental support and control variables

When assessing each form of parental support behaviour separately, all emerged as positively associated with adolescents’ MVPA in crude and partially adjusted analyses. However, in the fully adjusted model, only parental support in the form of encouragement and providing transportation to sport and physical activities were associated with 0.12 and 0.25 more days of MVPA per week, respectively. In direct comparisons by Wald test with other coefficients, the slope representing the fully adjusted association of transportation with MVPA emerged as a stronger correlate of MVPA than other parental behaviours, with the exception of parental encouragement and watching, which had equivalent levels of association (Table 3). Of all interaction terms tested, the intangible support by age interaction emerged as significant (*P* = 0.023), indicating that combined positive associations of parental support in the form of modeling, encouragement and informational increased with increasing age. Since other interaction terms were not significant, analyses suggest that the estimated associations of most parental support and control behaviours were consistent throughout adolescence in this cohort.

In analyses comparing the specific parental control behaviours, neither nagging nor ordering was significantly associated with MVPA in any iteration of the models.

**Fig. 1:**
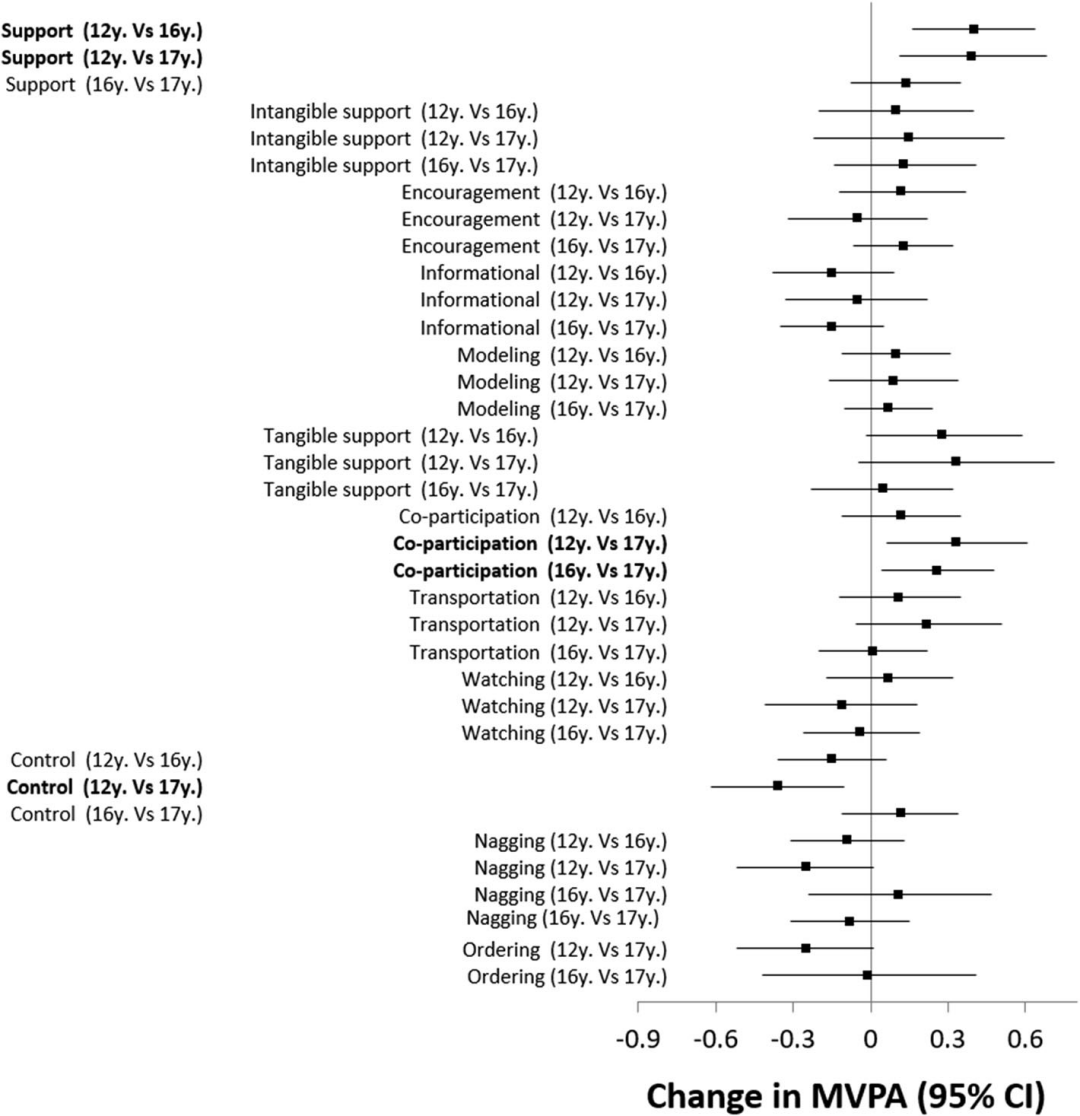
Estimated effects of combined and individual forms of parental support and control on adolescent’s future number of days for which they report at least 60 min of moderate to vigorous physical activity (*MVPA*). Terms in bold are those with *p* < 0.05. Analyses are adjusted for gender, urban/rural, neighborhood income and language of participant. Note: crude, partially adjusted and fully adjusted models all led to similar results so only fully adjusted models are presented

Overall parental support in early adolescence on their offspring’s MVPA appeared to last several years. Specifically, overall perceived parental support reported around age 12 was positively associated with MVPA measured 4 and 5 years later (Fig. 1). However, neither tangible nor intangible forms of parental support in early adolescence emerged as independent predictors of future MVPA in fully adjusted analysis. Of specific tangible parental support behaviours, only co-participation exerted an independent lagged association with MVPA 4 and 5 years later; none of the intangible parental support behaviours had notable lasting associations with MVPA.

The association of parental control in early adolescence with their offspring’s MVPA also appeared to be sustained over time. Specifically, perceived parental control reported around age 12 was negatively associated with MVPA measured 5 years later. There was no association between the specific control behaviours and MVPA.

## Discussion

This longitudinal study identified that adolescents reporting higher levels of perceived parental support also report higher levels of MVPA. Specifically, tangible forms of support, and especially providing transportation, had greater associations with MVPA than intangible forms of support. In contrast, higher perceived parental control was a negative predictor of MVPA. Further, we identified that perceived parental support and control reported during early adolescence had long lasting associations with MVPA up to 5 years later.

Our results align with previous reports of a positive association between general parental support and PA among youth [8, 11, 12]. Specifically, our results suggest that tangible parental support behaviours might have a more important positive effect on youth PA than intangible support. This may be because adolescents may face barriers towards PA if their basic tangible needs are not met [30]. Nonetheless, our results contrast those of studies reporting stronger associations between intangible support and PA than tangible parental support behaviours [31]. This may be due to previous studies not accounting for shared variance explained by the two types of support. For example, whereas our non-fully adjusted models pointed to positive associations of intangible parental support with adolescents’ MVPA, these associations dissipated in fully adjusted models. These results align with those of *Huffmann* et al. [32] who adjusted their analyses for different forms of support and found that tangible support from parents was positively associated with adolescents’ PA. These findings highlight the importance of considering the overlap of multiple parental support types when assessing the association between parental practices and PA.

We explored independent associations between several specific types of parental support behaviours and MVPA. In the literature, encouragement from parents is the most extensively studied parental support behaviour and fosters positive engagement of adolescents in PA [8]. Results from the current analysis support this statement, since encouragement was positively associated with MVPA which aligns with the suggestion that parents’ encouragements may exert a positive influence on MVPA through a higher perception of competency among their children [16]. Another tangible form of support that was a positive predictor of MVPA was transportation, which is consistent with studies summarized in a meta-analysis [12]. Providing transportation might be a marker for parental involvement by removing barriers to PA [16]. This may be particularly applicable for organized physical activities, which may represent up to 70% of adolescents leisure time PA [33]. This concurs with results from *Heitzel* et al. who documented higher odds of participation in organized PA among 9–13 year-olds who reported having access to transportation from parents [34]. Informational support was not identified as a predictor of MVPA and data scarcity among adolescents do not allow comparisons with other studies with similar samples [9]. However, a parallel can be made with a study conducted among pre-school-aged children (3–5 y), which found only a weak association between informational parental support and active play (r = 0.16, *P* = 0.02) [35]. Further, parental co-participation in PA was not statistically associated with MVPA. It is possible that considering the types of activities adolescents and their parents take part in (i.e. individual- or team-based) could have changed this association, so future studies should consider accounting for these additional factors.

In our study, parental control was negatively associated with MVPA. Results in the literature are mixed regarding associations between parenting styles and PA [17, 18]. Whereas one study reported that children raised in authoritative home environments (e.g. high parental control) may be more physically active [20], our results align with those of *Saunders* et al. who reported that Australian children (*n* = 919, 10–12 y) with more authoritative parents report lower levels of walking and cycling [36]. Such associations may be culturally dependent [18], but self-determination theory suggests that high parental control may lower the feeling of autonomy, which refers to the need for feeling that actions undertaken are volitional and emanate from personal decision [37]. Reduced perceptions of autonomy may then reduce motivation for PA and be associated with lower PA [38]. Nagging is one parental control behaviour studied herein and was negatively associated with MVPA. This highlights the need for parents to avoid the adoption of an insistent attitude towards the PA of their adolescents.

Overall, interactions showed that associations between parental support and MVPA seems to be stable across adolescence. Nonetheless, detailed assessments showed that the positive associations of intangible support with MVPA tends to increase as teenagers age. As adolescents become older, it may therefore be advisable for parents to encourage them when they are active, to provide them with information on PA and to themselves model engagement in PA.

A novel finding of this study was that even if the amount of perceived parental support declined over time, perceived parental support provided in early adolescence (12y.) had lasting associations with adolescents’ MVPA. In contrast, perceived parental support provided in late adolescence (16y. old) was not associated with MVPA 1 year later. Similarly, perceived parental control in early adolescence had lasting negative associations with teenager MVPA. Considering previous findings that parental influence decreases during adolescence and that peers or teachers exert greater influence than parents at the end of adolescence [38], our results suggest that establishing strong parental support in the foundational years of youth development might be key to sustain PA in the future. In particular, it was the effect of parental co-participation during early adolescence that seemed to most strongly affect MVPA 5 years later. More longitudinal research is required to assess optimal timing and amount of parental support as youth age.

Strengths of this study include the assessment of different forms of parental support and control in association with PA, analyses accounting for potential overlap among the effects of various parental behaviours, and multiple cycles of data collection throughout adolescence. The evaluation of a potential lagged association of parental behaviours on youth’s level of physical activity using a cross-lagged panel model was also a strength of this study. However, this approach does not account for the potential of unmeasured confounding and may not offer a complete protection against reverse causation bias. *Leszczensky and Wolbring* recently shown that a maximum likelihood SEM approach specifying both contemporary and lagged effects of the key independent variables could protect from the reverse causality [39]. Other limitations include the use of a self-reported measure of MVPA and perceived parental support. Our measure only included one dimension of parenting styles (control) and therefore does not inform on the association of permissive or uninvolved parenting on adolescents’ MVPA. Notwithstanding our self-reported measure, perceived parental support may have more importance than actual support offered as it will positively influence youth PA, which in turn improves youth self-efficacy beliefs and enjoyment of PA over time [15]. Finally, while it was recently reported that the average physical activity level of participants in the MATCH study is similar to the level observed among representative samples of Canadian youth [40], the study results may not be generalizable to all children and adolescents.

## Conclusion

This study highlights the importance of parental support as a determinant of youth PA. Although these implications need to be confirmed in experimental studies, parents could positively influence PA levels of their offspring throughout adolescence with tangible support behaviours, including co-participation and transportation, and intangible support behaviours such as providing encouragement. Given the sustained and lagged effects of parental support behaviours on youth MVPA, our results also highlight the importance of encouraging parental support behaviours in early adolescence. Finally, because of its negative association with MVPA, the control behaviours of parents, such as nagging, with regards to their adolescents’ PA levels should be discouraged to instead foster positive and supporting messaging about PA.

## Data Availability

The datasets generated during and/or analysed during the current study are not publicly available to ensure confidentiality and that any secondary analyses correspond to the objectives of the research project but are available from the corresponding author on reasonable request.

## Abbreviations

PA: Physical activity
MVPA: Moderate and Vigorous Physical Activity
MATCH: Monitoring Activities of Teenagers to Comprehend their Habits
RMSEA: Root mean square error of approximation
CFI: Comparative fit index
TLI: Tucker-Lewis index
